# Single‐Cell RNA‐Seq Reveals Injuries in Aortic Dissection and Identifies PDGF Signalling Pathway as a Potential Therapeutic Target

**DOI:** 10.1111/jcmm.70293

**Published:** 2024-12-25

**Authors:** Yichi Han, Yongji Cui, Juli Liu, Dingchen Wang, Guoxiang Zou, Xin Qi, Jinxiu Meng, Xiaoran Huang, Haiwei He, Xin Li

**Affiliations:** ^1^ Department of Emergency Medicine, Guangdong Provincial People's Hospital (Guangdong Academy of Medical Sciences) Southern Medical University Guangzhou Guangdong China; ^2^ Department of Critical Care Medicine Chengdu Fifth People's Hospital (The Second Clinical Medical College, Afliated Fifth People's Hospital of Chengdu University of Traditional Chinese Medicine) Chengdu China; ^3^ Medical Research Institute, Guangdong Provincial People's Hospital (Guangdong Academy of Medical Sciences) Southern Medical University Guangzhou Guangdong China; ^4^ School of Medicine South China University of Technology Guangzhou Guangdong China

**Keywords:** aortic dissection, cell death, cellular heterogeneity, oxidative stress, senescence, single‐cell RNA sequencing

## Abstract

Aortic dissection (AD) represents a critical condition characterised by a tear in the inner lining of the aorta, leading to the leakage of blood into the layers of the aortic wall, posing a significant risk to life. However, the pathogenesis is unclear. In this study, scRNA‐seq was applied to cells derived from aortas of both AD and non‐AD donors (control) to unveil the cellular landscape. ScRNA‐seq data uncover significant cellular heterogeneity in AD aortas. Specifically, we observed an accumulation of CD4^+^ T cells, which contributed to inflammation and cell death, and abnormal collagen formation mediated by fibroblast cells in AD. Moreover, we revealed a greater prevalence of cell death, oxidative stress and senescence in AD aorta cells. Furthermore, we found a decrease in the percentage of vascular stem cells (VSCs), along with a repression in their ability to differentiate into contractile vascular smooth muscle cells (VSMCs). Finally, our data demonstrated that the PDGF signalling pathway was activated in AD. We found that PDGF activation could lead to VSMCs aberrant switch from contractile to synthetic phenotype, which could be ameliorated by PDGF inhibitor. This underscores the potential of the PDGF as a therapeutic target for AD. In summary, our study highlights the cellular heterogeneity and associated injuries within aortas affected by AD, including cell death, oxidative stress, senescence and dysregulation of signalling pathways influencing the aberrant phenotypic switch of VSMCs. These insights offer valuable contributions to understanding the molecular mechanisms underlying AD and present new avenues for therapeutic intervention in this condition.

**Abbreviations**
AD: aortic dissection
BCA: bicinchoninic acid
BSA: bovine serum albumin
CNN1: calponin1
ECs: endothelial cells
FBS: fetal bovine serum
OPN: osteopontin
PBS: phosphate buffer saline
PDGFB: platelet derived growth factor B
RIPA: radio immunoprecipitation assay
TBST: tris buffered saline with tween 20
TGF‐β1: transforming growth factor‐β1
VSCs: vascular stem cells
VSMCs: vascular smooth muscle cells
WB: western blotting
α‐SMA: alpha‐smooth muscle actin

## 1 Introduction

Aortic dissection (AD) is a medical emergency that occurs when the inner layer of the aorta, the large blood vessel that carries blood from the heart to the rest of the body, tears or separates from the middle layer [1]. Although various treatments are available, such as medical, percutaneous aortic intervention and surgical intervention, AD remains the leading cause of morbidity and mortality worldwide [2]. The pathogenesis of aortic dissection entails the loss of vascular smooth muscle cells (VSMCs) within the vessel wall, degradation of the extracellular matrix (ECM), collagen deposition and inflammation [3, 4, 5, 6, 7]. Research has demonstrated that, in the AD vessel wall, the number of VSMCs decreased and the remaining VSMCs had undergone phenotypic switching, in which VSMCs shift between a differentiated, contractile phenotype and a dedifferentiated, synthetic phenotype, the latter characterised by decreased expression levels of VSMCs marker genes, the increase in extracellular matrix component synthesis and the increase in VSMCs proliferation and migration on exposure to various stimuli [5, 8, 9, 10, 11]. This can occur due to a combination of factors such as high blood pressure, genetic disorders that affect connective tissue, atherosclerosis, trauma or other conditions that weaken the aortic wall [12]. Once the false lumen is created, blood can flow between the layers of the aortic wall, causing further tearing and rupture, which can lead to organ failure, stroke or aortic rupture. The exact mechanism of aortic dissection is complex and not fully understood, but it involves changes in the structure and function of the vessel wall, as well as alterations in the blood flow and pressure within the aorta.

Our previous investigations unveiled a reduction in vascular smooth muscle cells (VSMCs) derived from vascular stem cells (VSCs) in Marfan syndrome (MFS) patients with AD attributed to VSC senescence [13]. We aimed to assess VSC function isolated from MFS patients. VSCs were isolated and characterised from both control and MFS donors. In comparison with controls, MFS‐VSC numbers declined, while cellular aging escalated and the capacity to differentiate into VSMCs diminished [13]. Cell senescence primarily manifested through increased cell size, heightened SA‐β‐gal activity and elevated levels of p53 and p21 [13]. RNA sequencing outcomes indicated significant enrichment of MFS‐VSCs in various pathways related to cell processes such as the cell cycle and cell aging [13]. This implies that VSCs may play a pivotal role in replenishing VSMC numbers in AD. This suggested that VSCs may have a critical role in replenishing the number of VSMCs in AD and also suggested that heterogeneity in cell types might contribute to AD. Therefore, a more comprehensive understanding of cell type heterogeneity and more molecular mechanisms underlying VSCs differentiation into VSMCs is needed for developing new therapeutic strategies for early management of AD patients.

Some studies have demonstrated the existence of a population of resident VSCs in the aortic adventitia that express Sca‐1 and c‐kit (Sca‐1^+^ and c‐kit^+^ VSCs) [14, 15, 16]. These VSCs are critical for cardiovascular regeneration therapy, as they can migrate from the adventitia to the media or intima and differentiate into smooth muscle cells and endothelial cells to support the vessel [17, 18, 19]. Under normal conditions, they can maintain vascular homeostasis and participate in vascular repair after injury [20]. However, in certain pathological conditions, the number of VSCs decreases and their ability to proliferate, migrate and differentiate is weakened, preventing them from fulfilling their role in vascular repair [21, 22]. Our previous study found that the number of VSCs from Marfan syndrome patients was reduced and senescence of VSCs was also increased compared to cells from health donors [13]. Additionally, the induced differentiation of these cells into VSMCs resulted in decreased expression of markers associated with contractile VSMCs, suggesting the VSMCs shift from a differentiated, contractile phenotype (contractile VSMCs [cVSMCs]) to a dedifferentiated, synthetic phenotype (synthetic VSMCs [sVSMCs]). However, the mechanisms are still unclear.

Single‐cell RNA sequencing (scRNA‐seq) is a powerful technique that allows for the analysis of gene expression in individual cells. The heterogeneity and complexity among cells are revealed by single‐cell sequencing technology and provides a unique perspective and approach for studying diseases. In the field of disease investigation, single‐cell sequencing technology assists in identifying new cell types, discovering rare cell populations, understanding cellular states and phylogenetic maps, thereby enhancing our comprehension of disease pathogenesis. Here, we performed scRNA‐seq of aortas from AD patients and non‐AD donors. Finding the accumulation of CD4^+^ T cells, aberrant collagen accumulation, oxidative stress and senescence in AD aortas‐derived cells. Additionally, we also found that a signalling pathway mediated by PDGFB (platelet‐derived growth factor subunit B) is involved in the VSMCs switch from a contractile phenotype to a synthetic phenotype. Our study suggested that targeting the PDGF signalling pathway could be a potential therapeutic approach for AD.

## 2 Materials and Methods

### 2.1 Isolation, Purification and Differentiation of VSCs

Vascular stem cells were isolated from control donors and patients with type A aortic dissection at the People's Hospital of Guangdong Province, China. All human samples shared a common age range of 30–72 years, and all participants exclusively had hereditary connective tissue disorders or genetic diseases, such as Marfan syndrome. The AD patients in our study were individuals who underwent aortic replacement surgeries at our hospital due to aortic rupture resulting from acute dissection (AD) disease. The term ‘Control donors’ refers to healthy individuals who unfortunately lost their lives under various circumstances, such as car accidents. Patient information is shown in Table 1. Written informed consent has been obtained from all study patients. This study was approved by the Research Ethics Committee of Guangdong Provincial People's Hospital (No. KY‐Z‐20210219‐02).

**TABLE 1:** Patient information.

	Sex	Age (year)	Diagnosed MFS	Reoperation
AAD1*	Male	67	No	No
AAD2*	Male	56	No	No
AAD3*	Female	72	No	No
AAD4	Male	67	No	No
AAD5	Female	58	No	No
AAD6	Male	67	No	No
Control 1*	Male	38	No	No
Control 2*	Female	36	No	No
Control 3	Male	57	No	No
Control 4	Female	54	No	No
Control 5	Male	60	No	No

Abbreviations: AD, aortic dissection; MFS, Marfan Syndrome.

* The highlighted samples denoted for single‐cell RNA sequencing (scRNA‐seq); Control 1 and Control 2 originate from the heart transplant donor, while the remaining samples are sourced from the heart transplant recipient.

#### 2.1.1 Isolation

Following the removal of adipose tissue, a primary cell culture was conducted using aortic adventitia. The tissue was dissected and processed into a single‐cell suspension using 1% collagenase type 1 (5401020001, Roche) for 180 min at 37°C. The cell suspension was then washed three times in 1× PBS and filtered through a 100 μm sieve before being centrifuged and plated into culture flasks. The cells were cultured in F‐12 medium supplemented with 15% fetal bovine serum, 10 ng/mL leukaemia inhibitory factor (lif1010, Millipore), and 1% penicillin streptomycin sodium at 37°C and 5% carbon dioxide.

#### 2.1.2 Purification

To obtain a purified population of c‐kit^+^ VSCs, positive cell sorting was performed using c‐kit (CD117) magnetic beads (130‐091‐332, Miltenyi). The cells were selected at passages 3–5 for subsequent assays.

#### 2.1.3 Differentiation

Characterisation of adipogenic, osteogenic and chondrogenic differentiation was carried out using the human mesenchymal stem cell function assay kit (SC006, R&D Systems). To induce smooth muscle cells (VSMCs) differentiation, TGF‐β1 (10 ng/mL) was utilised.

### 2.2 Characterisation of VSCs

Flow cytometry was utilised to evaluate the surface markers of Control‐VSCs and AD‐VSCs. Antibodies against various markers including anti‐c‐kit (Biolegend, 313205), anti‐CD29 (Biolegend, 303003), anti‐CD73 (Biolegend, 344003), anti‐CD105 (Biolegend, 323205), anti‐CD90 (Biolegend, 328107), anti‐CD31 (Biolegend, 303111) and anti‐CD45 (Biolegend, 304011) were used in the analysis.

### 2.3 Western Blotting

The concentration of each sample was determined using a bicinchoninic acid assay kit (231227, Thermo) after extracting protein from various treatments using RIPA buffer (9806, CST). Twenty micrograms of protein was separated on SDS‐PAGE gel and transferred to PVDF membranes from each sample. The membranes were washed thrice with Tris‐buffered saline containing 0.1% Tween‐20 (TBST), blocked with TBST containing 5% fat‐free milk and incubated overnight at 4°C with primary antibodies, including anti‐PDGFB (1:1000, ab178409, Abcam), anti‐Calponin1 (1:5000, ab46794, Abcam), anti‐a‐SMA (1:5000, ab124966, Abcam) and anti‐GAPDH (1:10000, 60004‐1, Proteintech). Subsequently, the membranes were rinsed thrice with TBST and incubated with secondary antibodies at room temperature for an hour. Finally, the membranes were exposed to enhanced chemiluminescence (ECL plus) (Amersham) in a dark environment.

### 2.4 Immunofluorescence Staining

Cells of different groups were fixed using 4% paraformaldehyde for 15 min. Following permeation with 0.1% Triton X‐100 in PBS for 30 min. Subsequently, the cells were incubated overnight at 4°C with the following primary antibodies: anti‐c‐kit (1:1000, ab32363, Abcam), anti‐Calponin1 (1:250, ab46794, Abcam), anti‐α‐SMA (1:2500, ab124964, Abcam), anti‐Osteopontin (1:100, ab63856, Abcam). After washing with TBST three times, the slides were incubated with fluorescent secondary antibodies (Abcam) for 60 min at room temperature. Finally, the sample was mounted with DAPI and photographed. Images of five different view fields for each slide were captured randomly by a motorised inverted microscope.

### 2.5 Phenylephrine Contraction Experiments

Cells with different treatments were seeded in six‐well plates. At about 60% cell density, the medium was changed to complete medium containing phenylephrine (1 μmol/mL). The cell size (area) changes at 0 and 30 min were observed under microscope, followed with quantification, respectively.

### 2.6 Single‐Cell Sequencing and Data Processing

#### 2.6.1 Processing Single‐Cell RNA‐Seq Data

In the study of human abdominal aortic aneurysm (AAA) samples (3 controls vs. 3 AAA patients), we processed single‐cell suspensions using a Single Cell Controller instrument (10× Genomics, USA) to generate gel beads in emulsions (GEMs). Subsequently, we constructed single‐cell RNA‐seq libraries utilising the Chromium Single Cell 3′ Library & Gel Bead Kit (10× Genomics, USA) as per the manufacturer's guidelines. These libraries were sequenced on an Illumina Novaseq 6000 platform. Raw sequenced data underwent processing via Cell Ranger software (10× Genomics, USA, version 3.0.2), where reads were aligned to the reference genome hg19 using the STAR aligner. Gene expression analysis, encompassing filtering, barcode counting, UMI counting and the generation of a gene count versus cells matrix, was executed using the cell count pipeline provided by Cell Ranger. Following mapping, six objects were created and subjected to quality control assessment using the Seurat package (version 4.1.0) in R.

#### 2.6.2 Quality Control

To ensure data reliability and accuracy, cells were further filtered based on predetermined threshold parameters: Genes expressed in fewer than 10 cells were excluded from subsequent analysis. Single cells expressing more than 300 genes while maintaining less than 20% mitochondrial gene content were retained. Only cells meeting these quality control criteria were included in downstream analyses. Normalisation procedures were conducted as per package instructions.

#### 2.6.3 Sample Correction and Integration

Utilising functions inherent to the Seurat packages, we corrected for batching effects and merged datasets. The FindIntegrationAnchors function facilitated the discovery of anchors among the six Seurat objects, which were subsequently used to integrate the objects employing the IntegrateData function.

#### 2.6.4 Dimensionality Reduction and Cluster Analysis

Nonlinear dimensional reduction of normalised gene expression values was performed to visualise cellular heterogeneity using the Seurat package. Initially, 2000 highly variable genes were identified for subsequent principal component analysis (PCA). Principal components (PCs) effectively segregating the cells were selected through jack straw analysis with 1000 replicates. Based on this analysis, the top 20 PCs were chosen for t‐SNE and UMAP dimensional reduction. The FindClusters function, with a resolution of 0.5, facilitated the identification of 12 distinct cell types.

#### 2.6.5 Identification of Differentially Expressed Genes

Differential gene expression analysis across identified cell types was conducted using the Seurat FindAllMarkers and FindMarkers functions, leveraging normalised gene expression values. By comparing gene expression profiles across different cell types, unique cell‐type‐specific marker genes were identified. Gene Ontology (GO) analysis was performed by the ClusterProfiler package. GO terms with Benjamini‐Hochberg adjusted *p*‐value < 0.05 were significantly enriched. GO analysis was utilised to explore the potential functions and crucial pathways. KEGG (Kyoto Encyclopedia of Genes and Genomes) analysis was also run on KEGG mapping tools (https://www.genome.jp/kegg/mapper/).

### 2.7 Cell Type Annotation of Vascular Smooth Muscle Cells

Particularly, the classification of subpopulations of VSMCs was mainly based on some published references. The contractile‐VSMCs are specifically expressed in some famous contractile genes such as MYH11, ACTA2, CNN1, TAGLN, MYLK and MYL9 while the synthetic‐VSMCs are associated with the synthesis of ECM genes such as MYH10, COL3A1, COL1A2, COL15A1, FN1 and COL8A1 [23, 24, 25]. However, unlike macrophage‐like‐VSMCs previously reported, the inflammatory‐VSMCs are highly expressed in some inflammatory genes such as complement genes C7, CC chemokine ligand CCL8, CCL19 and CCL21, as well as chemokine CXCL12. What is more, their GO analysis consequences exhibit the functions corresponding to their signatures.

### 2.8 Statistical Analysis

The mean and SE of the mean (SEM) are used to present the data from this experiment. Statistical analysis was conducted using GraphPad Prism 9. The Student's *t*‐test is used to compare the means between two independent groups, whereas one‐way ANOVA is used to compare the means among three or more groups. Results with a *p*‐value < 0.05 were considered statistically significant.

## 3 Result

### 3.1 Single Cell Transcriptomic Analysis of Thoracic Aorta Cells Derived From AD Patients and Controls (Non‐AD Donors)

**FIGURE 1:**
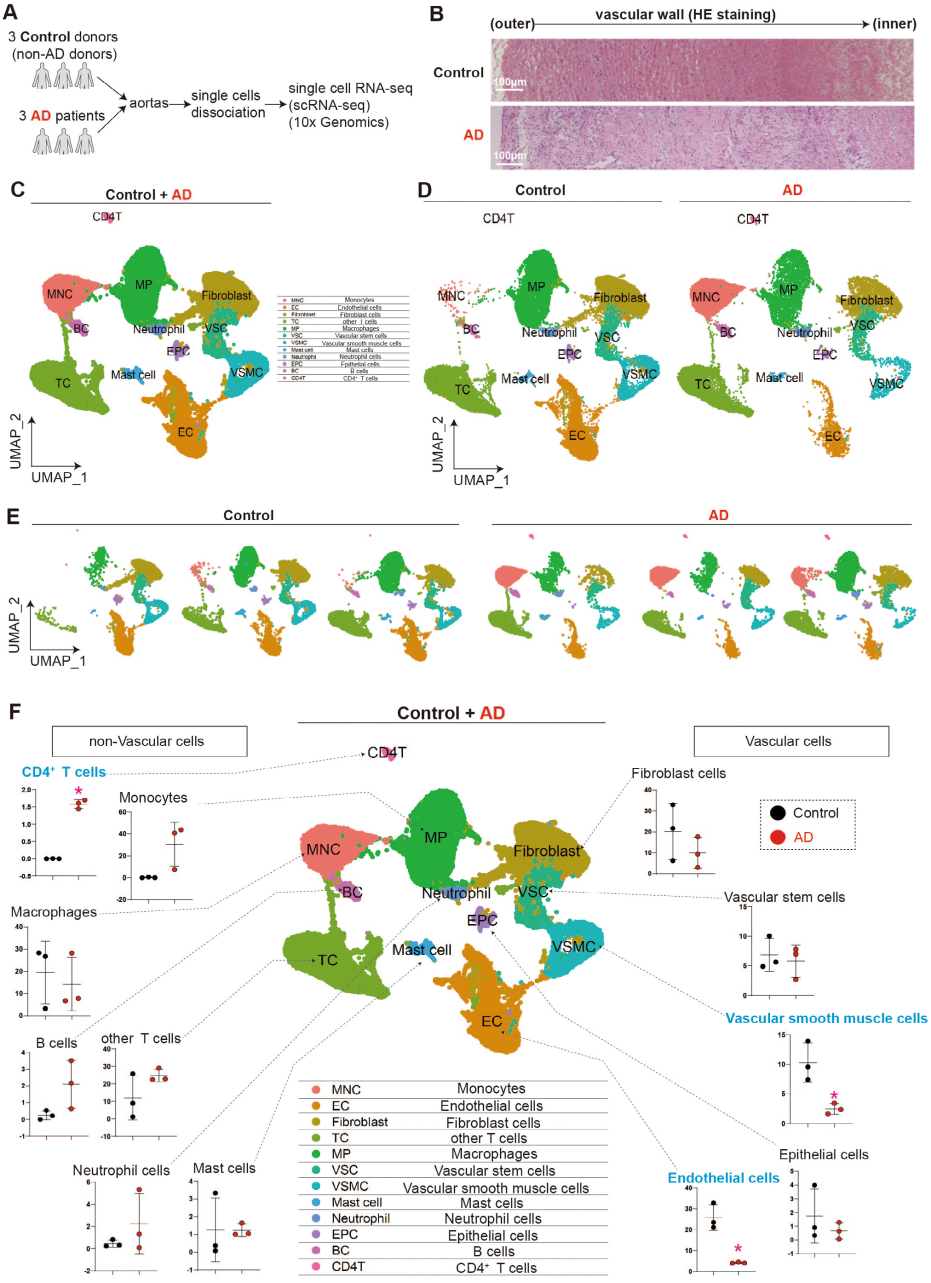
Single‐cell transcriptome analysis of thoracic aorta cells derived from AD patients and non‐AD donors. (A) Scheme of single‐cell RNA sequencing (scRNA‐seq) of thoracic aorta cells derived from aortic dissection (AD) patients and non‐AD donors. A total of three AD patients and three non‐AD donors were included in the study. (B) HE staining of aortas collected from AD patient and non‐AD donor (Control). (C–E) Uniform Manifold Approximation and Projection (UMAP) to analyse and visualise the cell clusters after integrating the scRNA‐seq data from the three control and three AD groups. (F) The percentages of different cell types in the three control and three AD groups determined by gene expression profiling. The cell types were classified as non‐vascular cells (including CD4^+^ T cells, monocytes, macrophages, other T cells, B cells, neutrophil cells and mast cells) and vascular cells (including fibroblast cells, vascular stem cells, vascular smooth muscle cells, epithelial cells and endothelial cells). *p < 0.05 (AD vs. Control), *N* = 3.

In this study, we utilised single‐cell RNA sequencing (scRNA‐seq), a robust technique that enables analysis of gene expression in individual cells [26], to investigate the pathogenesis of aortic dissection (AD) (Figure 1A). Specifically, scRNA‐seq was performed on cells obtained from the ascending aorta of both AD patients and non‐AD patients (control) (Figure 1A,B and Table 1). Uniform Manifold Approximation and Projection (UMAP) method [26] revealed distinct cell types, generally including non‐vascular cells (such as T cells) and vascular cells (such as endothelial cells) (Figure 1C–E). By comparing the proportion of specific cell types between control and AD aortas, we observed an increase in CD4^+^ T cell accumulation in AD aortas, as well as a reduction in the percentage of both vascular smooth muscle cells (VSMC) and endothelial cells (EC) (Figure 1F). As a result, our scRNA‐seq data provided helpful insights into the cellular heterogeneity involved in AD pathogenesis.

### 3.2 ScRNA‐Seq Reveals That the Accumulation of CD4 ^+^ T Cells Is Linked to Inflammatory Response and Cell Death in AD Aortas

**FIGURE 2:**
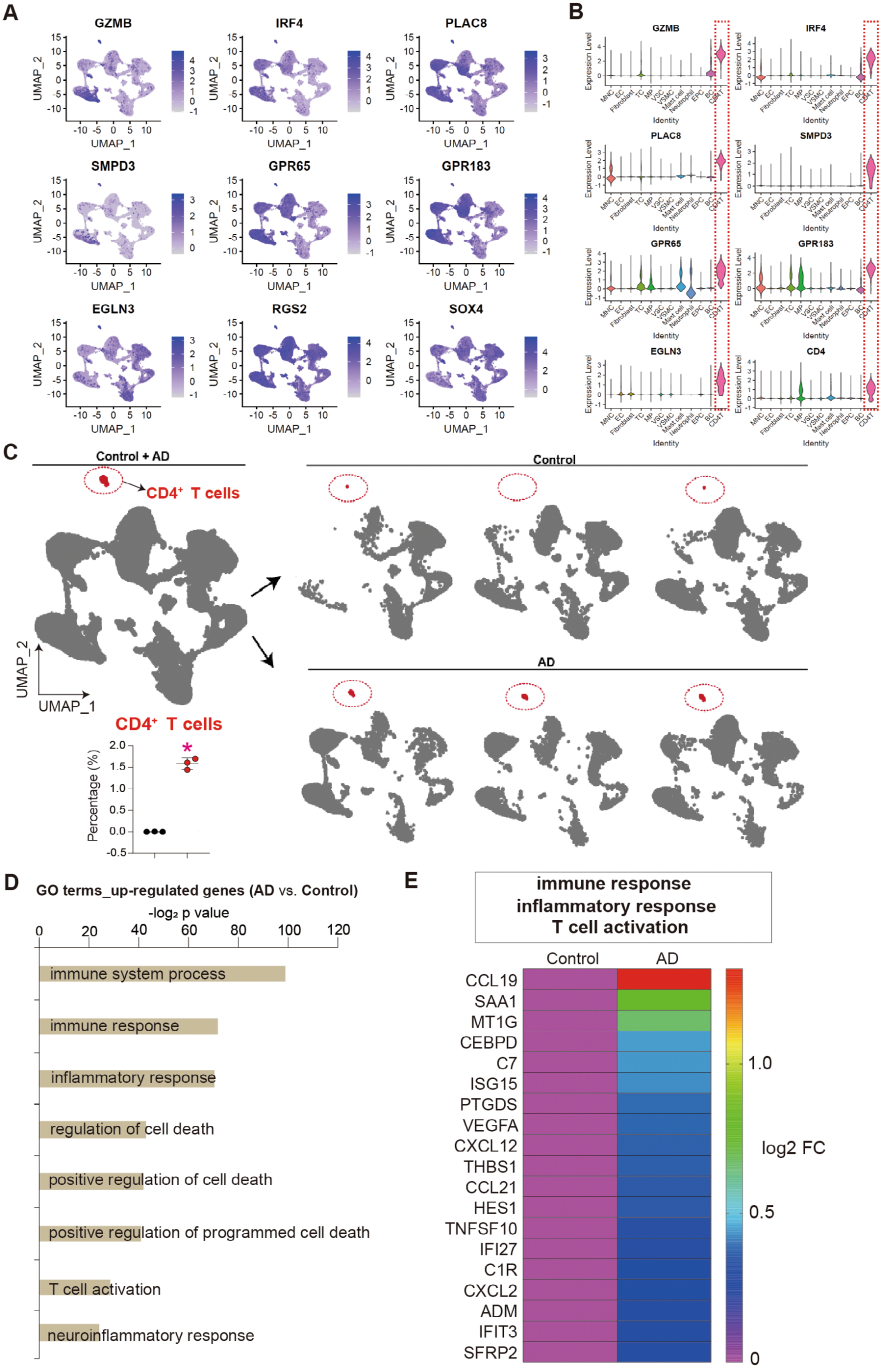
ScRNA‐seq reveals that the accumulation of CD4^+^ T cells is highly associated with inflammatory response and cell death in AD aortas. (A) UMAP analysis showing expression levels of CD4^+^ T cell markers. (B) Violin plots showing expression levels of CD4^+^ T cell markers. (C) UMAP analysis showing the CD4^+^ T cells. Red circle indicated CD4^+^ T cell cluster. (D) Gene ontology (GO) analysis of up‐regulated genes in AD aortas (AD vs. Control). Top GO terms were selected. (E) Heat map showing the up‐regulated genes inducing immune response, inflammatory and T cell activation. FC, fold change.

The scRNA‐seq revealed a significant increase in the percentage of CD4^+^ T cells in AD aortas compared to that in control aortas, albeit at a low percentage (approximately 1.5%) (Figure 1F). This increase in CD4^+^ T cells was associated with elevated expression levels of T cell markers, as well as the CD4 gene (Figure 2A,B). The integrative analysis demonstrated clear evidence of CD4^+^ T cell accumulation in AD aortas (Figure 2C). The up‐regulated genes in AD aortas were found to activate T cells, resulting in inflammatory responses and cell death (Figure 2D). Furthermore, the heat map displayed the activation of genes that promote inflammation (Figure 2E). We conducted subcellular reclustering and signalling pathway enrichment analysis of CD4^+^ T cells (Figure S1A–D), indicated that CD4^+^ T cells from aortic dissection patients may contain Tfh cells, Th22 cells and Th1 cells and might be linked to the differentiation or activation of pDCs. This suggested a potential connection between these cell types, pDCs differentiation or activation and the progression of aortic dissection, which warranted further investigation. And also suggested that the accumulation of CD4^+^ T cells was highly correlated with the inflammatory response and cell death in AD aortas, which indicated that activated CD4^+^ T cells may be responsible for inducing injuries in AD aortas.

### 3.3 ScRNA‐Seq Reveals Aberrant Collagen Accumulation in AD Aortas

**FIGURE 3:**
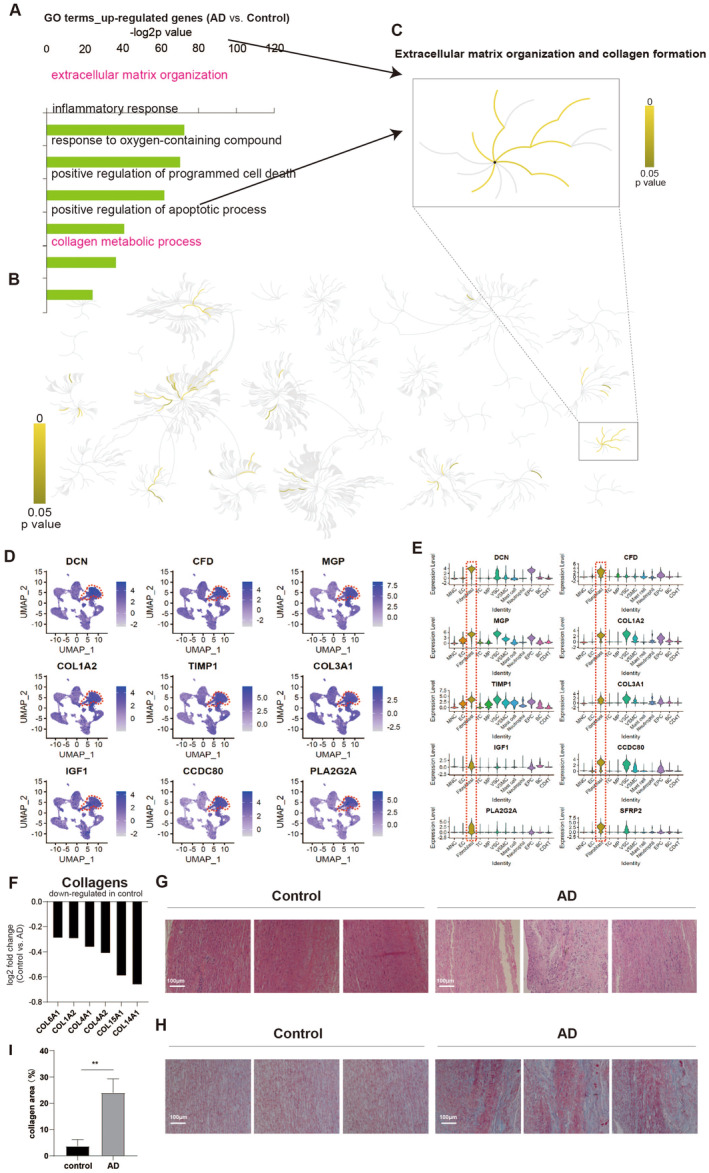
ScRNA‐seq reveals aberrant collagen accumulation in AD aortas. (A) GO analysis of up‐regulated genes in AD aortas (AD vs. Control). Top GO terms were selected. (B) Signalling pathway analysis of up‐regulated genes in AD aortas (AD vs. Control). Top Signalling pathways were highlighted with yellow with *p* value < 0.05. (C) Signalling pathway analysis showing collagen accumulation in AD aortas. (D) UMAP analysis showing expression levels of fibroblast cell markers. (E) Violin plots showing expression levels of fibroblast cell markers. (F) Expression levels of collagen markers in aortas. Y‐axis indicates the log_2_ fold change (AD vs. Control). (G) Haematoxylin and eosin (HE) staining of aortas sections from AD and non‐AD (Control). (H–I) Masson staining and quantitatively analyse of aortas sections from AD and non‐AD (Control). Blue colour indicated collagens.

The genes that are upregulated in aortas affected by AD have been found to promote extracellular matrix synthesis and collagen accumulation, as well as cell death and apoptosis (Figure 3A–C). Through an integrative analysis, it was determined that fibroblast cell populations were responsible for mediating collagen formation (Figure 3D,E). The expression levels of multiple collagen genes were significantly increased in AD‐affected aortas compared to control aortas (Figure 3F). Additionally, staining of aortic sections revealed increased collagen accumulation in AD patients (Figure 3G,H). Consequently, our study demonstrated that abnormal collagen accumulation occurred in the aortas of AD patients, which may, at least in part, explain the increased stiffness observed in AD aortas [27, 28].

### 3.4 ScRNA‐Seq Reveals Injuries in Vascular Stem Cells Derived From AD Aortas

**FIGURE 4:**
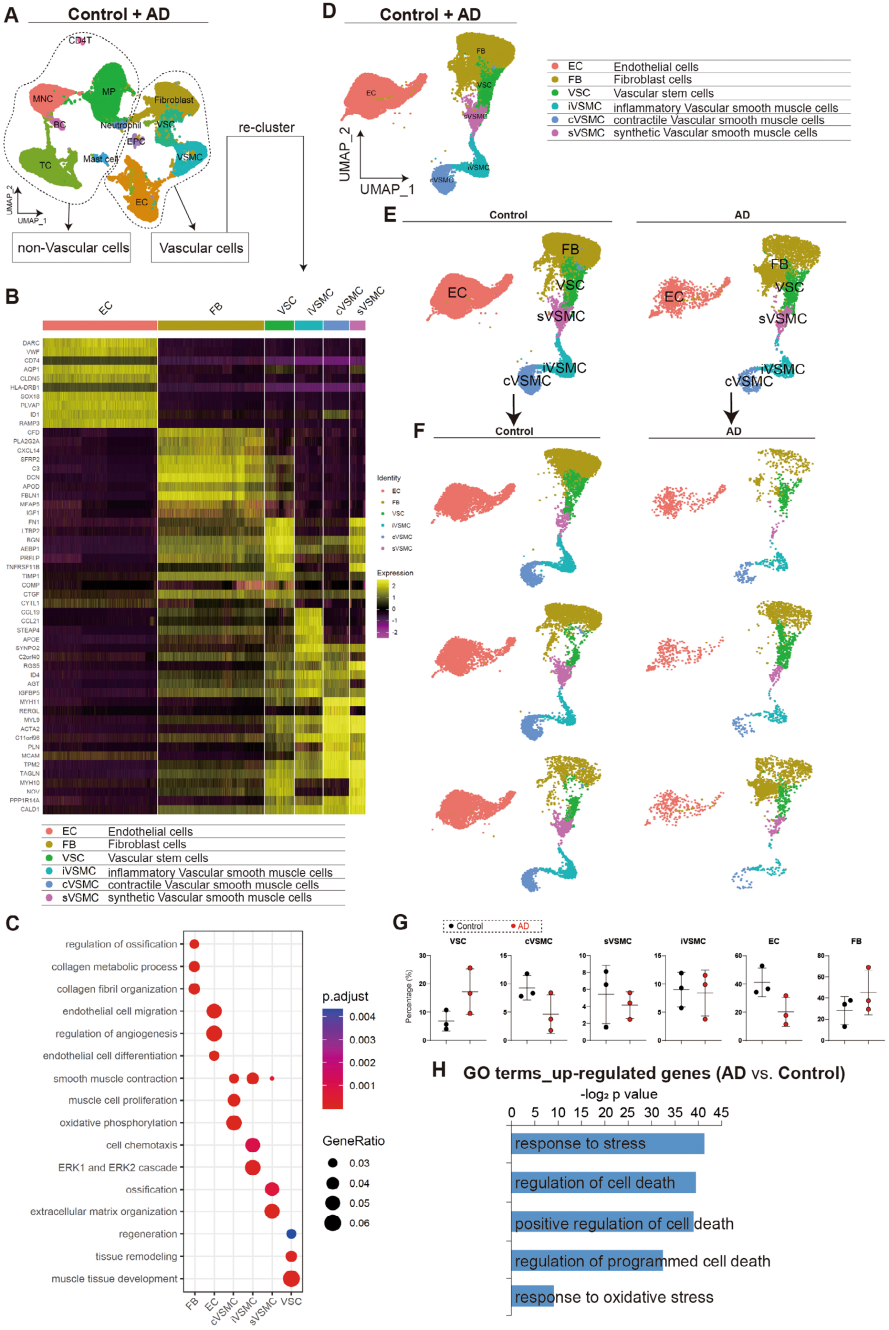
ScRNA‐seq reveals injuries in vascular cells derived from AD aortas. (A, B) Re‐clustering of scRNA‐seq based on gene expression profiling for vascular cells. (C) GO analysis of differentially expressed genes in different cell types of vascular cells. Top GO terms were selected. (D–F) UMAP analysis showing different cell types after re‐clustering. (G) Cell percentage of different cell types in control and AD. (H) GO analysis of up‐regulated genes in AD cells versus control cells. Top GO terms were selected.

Studies have shown that the number of vascular smooth muscle cells (VSMCs) in the AD vessel wall decreases and the remaining VSMCs undergo a phenotypic switch between a differentiated, contractile state and a dedifferentiated, synthetic state [5, 8, 9, 10, 11]. As a result, we first focused on various vascular cell types, such as fibroblasts, endothelial cells, vascular stem cells, vascular smooth muscle cells and epithelial cells. To investigate further, we re‐clustered scRNA‐seq data of aortic cells from both control and AD patients (Figure 4A), and identified six distinct cell populations based on their gene expression profiling (Figure 4B) and cellular function (Figure 4C): fibroblast cells (FB), endothelial cells (EC), vascular stem cells (VSC), synthetic vascular smooth muscle cells (sVSMC), contractile vascular smooth muscle cells (cVSMC) and inflammatory vascular smooth muscle cells (iVSMC) (Figure 4D–F). Although there was no significant change in the percentage of different cell types between the control and AD aortas (Figure 4G), we found up‐regulated genes in AD aortas that induced cell death and that AD aortic cells had higher levels of oxidative stress (Figure 4H).

**FIGURE 5:**
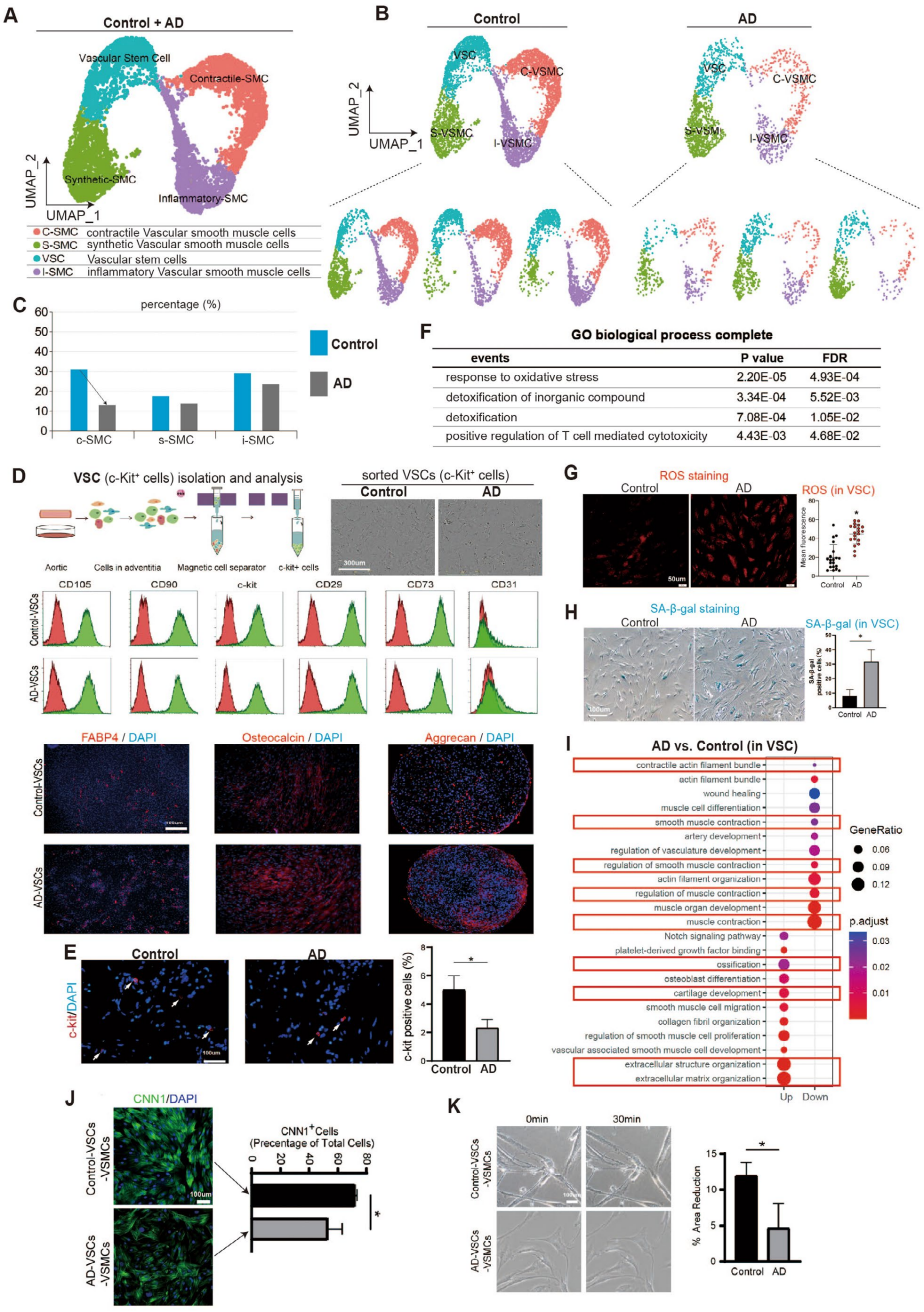
Vascular stem cells have higher oxidative stress and senescence, and inhibited differentiation towards contractile smooth muscle cell. (A, B) Re‐clustered scRNA‐seq data showing populations of vascular stem cells (VSCs), synthetic smooth muscle cells (sVSMCs), contractile smooth muscle cells (cVSMCs) and inflammatory smooth muscle cells (iVSMCs). (C) Percentage analysis showing decreased percentage of cVSMCs between control and AD. (D) Vascular stem cells (VSCs) isolation from cells in aortas of control and AD patients by using specific c‐Kit antibody. VSCs were further validated with different cell surface markers of VSCs. VSCs markers included CD105, CD90, c‐Kit, CD29 and CD73. CD31 was negative in VSCs. The VSCs isolated had multi‐potential differentiation capacity, including adipogenic (FABP4), osteogenic (Osteocalcin) and chondrogenic differentiation (Aggrecan). (E) C‐kit^+^ cell percentage in Control and AD aortas. *p < 0.05, *N* = 3. (F) GO analysis of up‐regulated genes in AD VSCs versus control VSCs. (G) ROS staining in control and AD VSCs. *p < 0.05. (H) SA‐β‐gal staining in control and AD VSCs. *p < 0.05, *N* = 3. (I) Top GO analysis of differentially expressed genes between AD VSCs versus control VSCs. (J) Immunostaining showing expression level of CNN1, a marker of contractile smooth muscle cells. Percentage of CNN1^+^ cells was quantified in control‐VSCs‐derived vascular smooth muscle cells (control‐VSCs‐VSMCs) and AD‐VSCs‐derived vascular smooth muscle cells (AD‐VSCs‐VSMCs). *p < 0.05, *N* = 3. (K) Cell size (area) quantification in control‐VSCs‐VSMCs and AD‐VSCs‐VSMCs. Cells were treated with or without phenylephrine. 30 min meant that cells were treated with phenylephrine for 30 min. The final concentration of phenylephrine was 1 μmol/mL. The size of cells treated with phenylephrine was quantified. *p < 0.05, *N* = 3.

Specifically, we investigated whether the VSMCs underwent a phenotypic switch between synthetic vascular smooth muscle cells (sVSMC) and contractile vascular smooth muscle cells (cVSMC), we continued to re‐cluster vascular cells in aortas (Figure 5A,B). Our finding indicated that the percentage of cVSMCs decreased in aortas affected by AD compared to control aortas (Figure 5C). This could be due to a decrease in the quality or differentiation of vascular stem cells (VSCs). Therefore, we isolated c‐Kit^+^ cells in both AD and control aortas, which were identified as VSC cells based on specific markers identified by flow cytometry and lineage specification capability (Figure 5D). Our results show that the percentage of c‐Kit^+^ cells was significantly lower in AD aortas than in control aortas (Figure 5E). Furthermore, we observed that the up‐regulated genes in AD‐VSCs compared to control‐VSCs induced oxidative stress (Figures 5F and S3), which was further validated by reactive oxidative species (ROS) staining in control and AD‐derived VSCs (Figure 5G). In addition, we detected a much higher expression level of SA‐β‐gal, a marker of senescence, in AD‐VSCs compared to control‐VSCs (Figure 5H). These findings provided evidence of greater senescence in AD‐derived vascular stem cells (VSCs) under oxidative stress.

We conducted a sequencing transformation trajectory analysis, as illustrated in Figure S2A. The data revealed differences in cell transformation between AD‐VSMCs and control‐VSMCs, as shown in Figure S2B. Detailed analysis based on gene expression profiles (see Figure S2C) indicated that AD‐VSMCs had a higher percentage of inflammatory and transitional SMCs, which may contribute to the progression of AD disease. We next investigated whether the differentiation of vascular smooth muscle cells (VSCs) into contractile smooth muscle cells (cVSMCs) was inhibited due to a decreased percentage of cVSMCs (Figure 5C). We performed KEGG signalling pathway analysis and found that genes up‐regulated in AD‐VSCs led to ossification, osteoblast and abnormal collagen accumulation in the extracellular matrix (Figure 5I), which was consistent with our previous findings (Figure 3). Conversely, down‐regulated genes in AD‐VSCs were associated with smooth muscle development and smooth muscle contraction (Figure 5I). These results suggested that the differentiation of AD‐VSCs towards VSMCs was inhibited and the contractile of VSMCs was repressed. To confirm this, we measured the percentage of CNN1^+^ cells, which are indicative of contractile VSMCs, and found that it was significantly lower in the AD group compared to the control group (Figure 5J). Additionally, we observed that AD‐derived contractile VSMCs exhibited significantly reduced contraction compared to control‐derived contractile VSMCs (Figure 5K).

**FIGURE 6:**
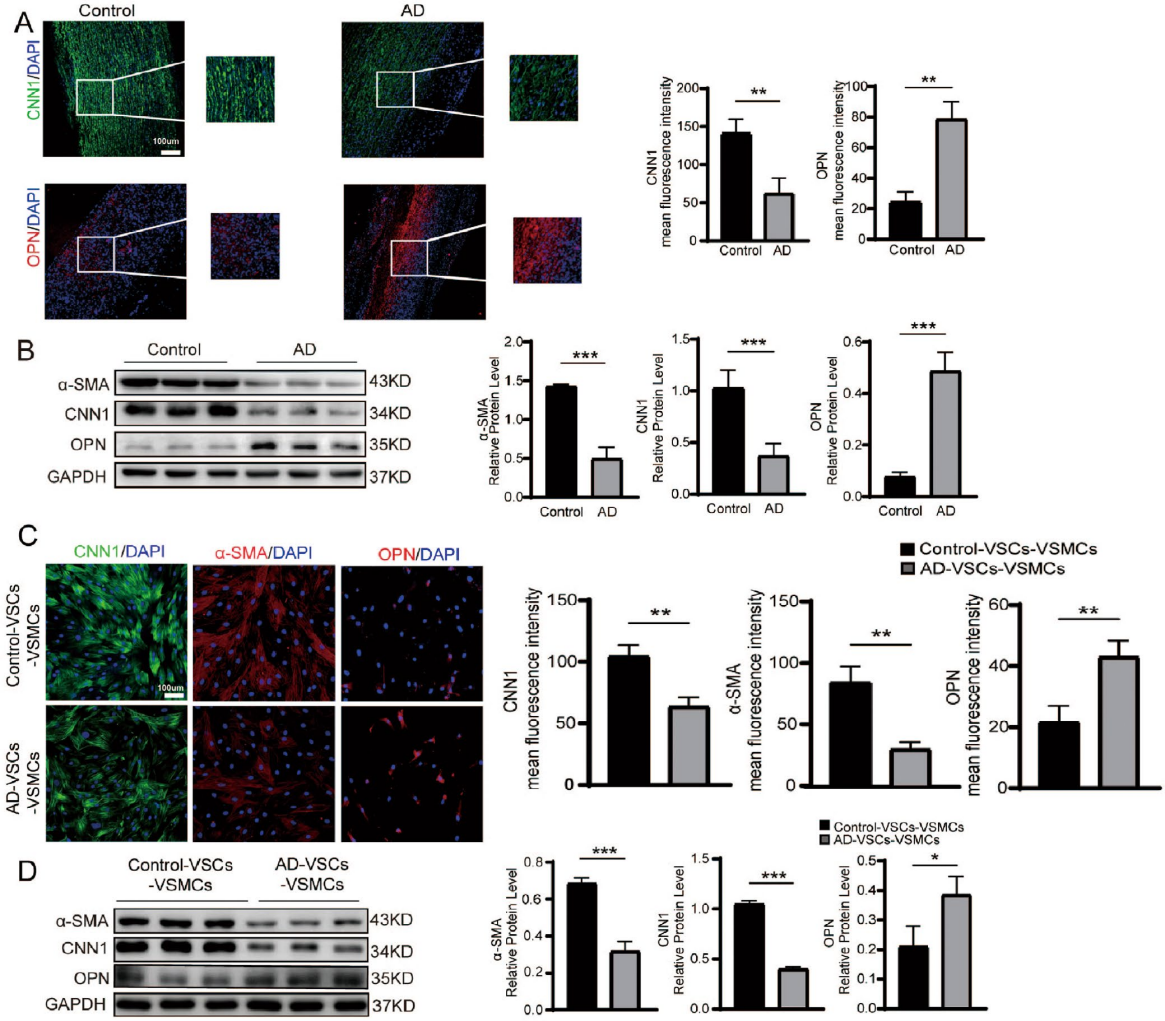
Smooth muscle cell switch from contractile phenotype to synthetic phenotype in AD aortas. (A) Immunostaining of CNN1 (contractile VSMC marker) and OPN (synthetic VSMC marker) in control and AD aortas. Mean fluorescence intensity of immunostaining was quantified. **p < 0.01, *N* = 3. (B) Western‐blotting detecting contractile VSMC markers (CNN1, α‐SMA) and synthetic VSMC marker (OPN) in AD and control aortas. ***p < 0.01, *N* = 3. (C) Immunostaining of CNN1 (contractile VSMC marker) and OPN (synthetic VSMC marker) in control‐VSCs‐VSMCs and AD‐VSCs‐VSMCs. Mean fluorescence intensity of immunostaining was quantified. **p < 0.01, *N* = 3. (D) Western‐blotting detecting protein expression levels of contractile VSMC markers (CNN1, α‐SMA) and synthetic VSMC marker (OPN) in control‐VSCs‐VSMCs and AD‐VSCs‐VSMCs. *p < 0.05, ***p < 0.01, *N* = 3.

We continued analysed the VSMC switch between the contractile VSMCs (cVSMCs) and the synthetic VSMCs (sVSMCs). We found that control aortas had higher expression level of CNN1 (cVSMCs marker) and lower expression of OPN (sVSMCs marker) indicated by immunostaining (Figure 6A). The western‐blotting data showed consistent results on the expression levels of CNN1 and OPN between control and AD groups (Figure 6B). We differentiated VSCs to smooth muscle cells and evaluated the switch between contractile and synthetic phenotypes in VSC‐derived smooth muscle cells (VSCs‐VSMCs) from AD and control groups. We found that expression levels of contractile‐associated markers (CNN1 and α‐SMA) were significantly decreased, whereas the expression level of synthetic associated marker (OPN) was significantly increased (Figure 6D).

Taken together, our data revealed injuries in vascular stem cells derived from AD aortas, including oxidative stress, senescence and the differentiation repression towards cVSMCs.

### 3.5 Platelet‐Derived Growth Factor (PDGF) Signalling Pathway Is Activated in AD and Promotes Smooth Muscle Cells Switch From Contractile Phenotype to Synthetic Phenotype

**FIGURE 7:**
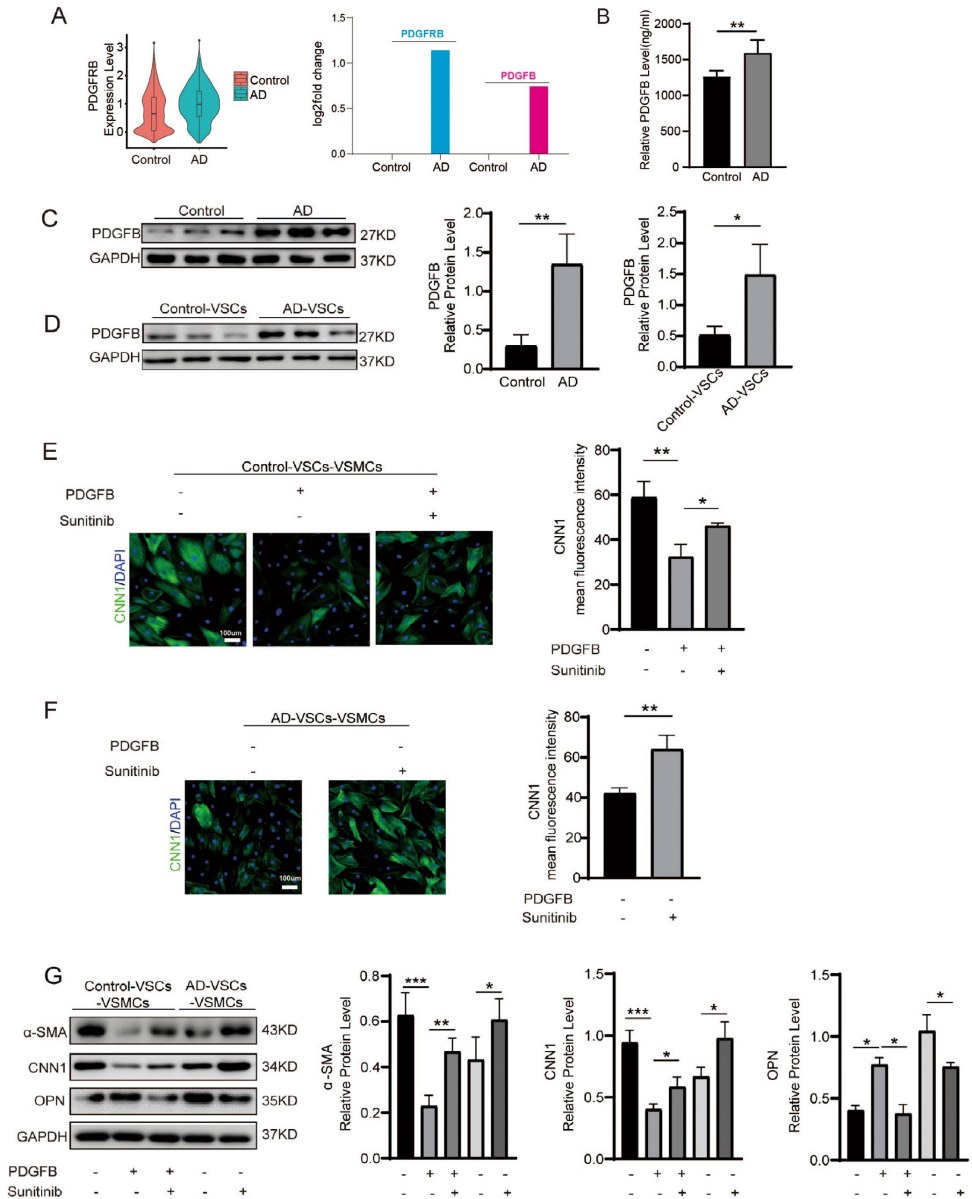
PDGF signalling pathway is activated and promotes smooth muscle cell switch from contractile phenotype to synthetic phenotype in AD aortas. (A) Violin plot showing PDGFRB expression level in Control and AD aortas (left). The expression changes of PDGFRB and its ligand PDGFB in Control and AD aortas (right). (B) ELISA detecting the expression level of PDGFB in serum from Control and AD patients. **p < 0.01, *N* = 3. (C, D) PDGFB protein expression level in aortas (C) and isolated VSCs (D). *p < 0.05, **p < 0.01, *N* = 3. (E) Immunostaining of CNN1, a marker of contractile VSMCs, in control‐VSCs‐VSMCs. Mean fluorescence intensity of immunostaining was quantified. *p < 0.05, **p < 0.01, *N* = 3. Sunitinib was an inhibitor of PDGFR signalling pathway. The final concentration of Sunitinib was 0.2 μM. DMSO was used as the control. (F) Immunostaining of CNN1, a marker of contractile VSMCs, in AD‐VSCs‐VSMCs. Mean fluorescence intensity of immunostaining was quantified. **p < 0.01, *N* = 3. (G) Western‐blotting detecting protein expression levels of contractile VSMC markers (CNN1, α‐SMA) and synthetic VSMC marker (OPN) in control‐VSCs‐VSMCs and AD‐VSCs‐VSMCs. Sunitinib was an inhibitor of PDGFR signalling pathway. The final concentration of Sunitinib was 0.2 μM. DMSO was used as the control. *p < 0.05, **p < 0.01, ***p < 0.001, *N* = 3.

Top GO analysis found that genes up‐regulated in AD‐VSCs led to activation of platelet‐drived growth factor binding signalling pathway (Figure 5I), indicating that platelet‐derived growth factor (PDGF) signalling pathway was activated in AD and might be involved in the pathogenesis of AD. To study the function of PDGF signalling pathway, we firstly evaluated the expression levels of PDGF and its receptor PDGFR. The results of ScRNA‐seq analysis revealed that the expression levels of PDGFRB (Platelet‐Derived Growth Factor Receptor Beta) and its ligand PDGFB (Platelet‐Derived Growth Factor, Subunit B) were elevated in AD, compared to control samples (Figure 7A). PDGFB protein level in serum from AD patients was upregulated (Figure 7B). Furthermore, PDGFB protein levels in AD aortas and AD‐VSCs were also upregulated (Figure 7C,D). These findings suggested that the PDGFR signalling pathway may be activated in cells from AD aortas. To explore this possibility, we investigated the effects of PDGFB and the PDGFR signalling pathway inhibitor Sunitinib [29] in cells from AD aortas (Figure 7E). We observed that PDGFB suppressed the expression of CNN1 in control‐VSCs‐VSMCs, which was restored by Sunitinib (Figure 7E). Similarly, in AD‐VSCs‐VSMCs, Sunitinib treatment increased CNN1 expression (Figure 7F). Consistent changes in VSMCs phenotypes were also observed in western‐blot data (Figure 7G). We found that expression levels of contractile markers (CNN1, α‐SMA) were down‐regulated by PDGFB and up‐regulated by Sunitinib, whereas expression levels of synthetic marker (OPN) were increased by PDGFB and decreased by Sunitinib (Figure 7G). Taken together, our findings demonstrated that platelet‐derived growth factor (PDGF) signalling pathway activated the smooth muscle cells switch from contractile phenotype to synthetic phenotype in AD.

## 4 Discussion

To shed light on these mechanisms in AD, we used single‐cell RNA sequencing (scRNA‐seq) to analyse the gene expression of individual cells from the aortas of both AD patients and non‐AD donors (controls). Our findings revealed a high level of cellular heterogeneity in both the aortas and injuries, including cell death, oxidative stress, senescence and dysregulation of signalling pathways that are involved in AD. We identified specific types of cells that were involved in causing injury, as well as the reasons behind the aberrant switch in phenotype of smooth muscle cells (VSMCs). Ultimately, we determined that the PDGF signalling pathway may hold potential as a therapeutic target for treating AD.

In the AD, a tear develops in the inner layer of the aorta, the main artery that carries oxygen‐rich blood from the heart to other organs in human. Blood can flow through the tear, separating the inner and middle layers of the aortic wall, causing a false channel called a ‘dissection’ to form [1]. Depending on the location and extent of the dissection, blood flow to various organs can be affected. As the dissection progresses, blood flow to various organs can be disrupted, potentially causing complications such as heart attack, stroke or organ damage. Aortic dissection is a medical emergency that requires immediate diagnosis and treatment to prevent serious complications or death. The pathological mechanism of AD involves a weakening of the aortic wall, often caused by degenerative changes in the tissue, genetic factors or conditions that increase blood pressure or strain on the aorta [5, 8, 9, 10, 11]. However, the underlying molecular mechanisms remain to be elucidated.

Single‐cell RNA sequencing (scRNA‐seq) is a powerful technique used to profile the gene expression of individual cells [26]. By analysing the transcriptomes of thousands of individual cells, scRNA‐seq can provide insights into the cellular composition and activity of complex tissues and organs, including the aorta [30, 31]. Recent studies have used scRNA‐seq to investigate the molecular mechanisms underlying aortic dissection. A study [30] used scRNA‐seq to analyse cells of aortic tissues from patients with ascending thoracic aortic aneurysm (ATAA) and identified specific cell types and signalling pathways that were dysregulated in ATAA. In the study, they found ATAA tissues had fewer nonimmune cells and more immune cells, especially T lymphocytes, than control tissues did. Differential gene expression data suggested the presence of extensive mitochondrial dysfunction in ATAA tissues [30]. However, this study mainly focused on immune cells (or non‐vascular cells), other than vascular cells. Differently, in our study, we focused on not only vascular cells (such as fibroblast cells, endothelial cells, vascular stem cells and smooth muscle cells) but also non‐vascular cells such as T cells. Our scRNA‐seq uncovered an accumulation of CD4^+^ T cells in AD aortas. Moreover, we found the accumulation of CD4^+^ T cells were highly associated with inflammation and cell death in AD aortas. This indicated that CD4^+^ T cells may promote AD, although the detailed function remains to be elucidated in future.

Recently, an Il1rn^+^/Trem1^+^ macrophage subpopulation was identified as a cellular target for mitigating the progression of thoracic aortic aneurysm and dissection by using scRNA‐seq [31]. This study showed that macrophages might be important for aortic dissection. Another study also found that Th17‐like cells might be important for AD [32]. In our study, we uncovered an accumulation of CD4^+^ T cells in AD aortas. These evidences showed that non‐vascular cells, such as macrophage and CD4^+^ T cells, were involved into the AD disease, which should be further investigated.

It was demonstrated that dysregulation of interaction between LOX^(high)^ fibroblast and smooth muscle cells contributed to the pathogenesis of aortic dissection [33]. We found abnormal collagen accumulation mediated by fibroblast cells in AD aortas as well. This indicated that fibroblast cells played an important part in AD. Collagen content and fibrosis were histologically examined in the wall proximal to the AD. It was reported that in this area the collagen content increased in the medial layer of AD compared with control. Also, increased fibrosis was seen in the wall of dissected tissue [34]. This could explain the increased stiffness observed in AD aortas [27, 28]. In our study, the scRNA‐seq data revealed that aberrant collagen formation and extracellular matrix synthesis were observed in the AD aortas, which was mediated by fibroblast cells. All these evidences demonstrated the critical function of fibroblast cells in AD pathogenesis. And this could, at least in part, explain the stiffness of AD aortas compared to that of healthy aortas.

Our previous study showed that the number of vascular stem cells (VSCs) from Marfan syndrome patients exhibited a decrease compared to that from Control donors [13]. This indicated that VSCs might also contribute to AD pathogenesis. Here, we isolated VSCs (c‐Kit^+^ cells), which had multi‐potential differentiation capacity, and found that percentage of VSCs was significantly decreased in AD aortas, compared to that of control aortas. In the AD VSCs, there were higher levels of reactive oxidative species (ROS) and SA‐β‐gal, showing more oxidative stress and senescence in AD VSCs. This might contribute to the decreased percentage of VSCs in AD aortas. Furthermore, we also uncovered that the differentiation of VSCs towards contractile smooth muscle cells (cVSMCs) was also repressed in AD aortas. This might also be, at least in part, attributed to the more oxidative stress and senescence in AD VSCs, which could lead to cell death.

Except that we found the differentiation of VSCs towards contractile smooth muscle cells (cVSMCs) was also repressed in AD aortas, our data further demonstrated that expression levels of contractile phenotype markers in VSMCs were repressed whereas the expression level of synthetic phenotype marker was activated in AD aortas. This meant that the VSMCs phenotype switch from cVSMCs to sVSMC was presented in AD, which was consistent with previous studies [5, 8, 9, 10, 11].

Importantly, by analysing the scRNA‐seq data, we observed that PDGFRB and its ligand PDGFB were significantly up‐regulated in AD aortas than that in control aortas. PDGFB have been implicated in the development of many pathological processes, such as cardiovascular disease. For example, serum PDGFB level was elevated in patients with hypertension and hypercholesterolemia [35, 36]. PDGFB can participate in the development of intimal hyperplasia and atherosclerosis after injury [35]. PDGFB might be involved into VSMCs dedifferentiation, proliferation and migration [37]. However, whether PDGFB could function in AD was still unclear. In our study, we found expression levels of both PDGFB and its receptor PDGFRB were significantly up‐regulated. This indicated that PDGF signalling pathway was activated in AD aortas. Therefore, we used PDGFB and PDGF pathway inhibitor Sunitinib as the tools to regulate PDGF signalling pathway in cells derived from AD aortas and control aortas. We found that PDGFB activated expression level of synthetic phenotype marker and repressed expression levels of contractile phenotype markers in VSMCs. Conversely, Sunitinib repressed expression level of synthetic phenotype marker and activated expression levels of contractile phenotype markers in VSMCs. This suggested that activated PDGF signalling pathway in AD led to VSMCs phenotype switch from cVSMCs to sVSMCs, which subsequently promoted AD pathogenesis. Our findings showed that PDGFR signalling pathway would be a therapeutic target for AD.

## 5 Conclusion

Taken together, our findings showed cellular heterogeneity of aortas and injuries, including cell death, oxidative stress, senescence and signalling pathway dysregulation involved in aortic dissection. We also identified that PDGFR signalling pathway may be a potential therapeutic target for AD. In conclusion, our findings have the potential to provide valuable insights into the molecular mechanisms of aortic dissection and to identify new therapeutic strategy for aortic dissection.

## Author Contributions

**Yichi Han:** formal analysis (equal), investigation (equal), writing – original draft (equal). **Yongji Cui:** investigation (equal). **Dingchen Wang:** writing – original draft (equal). **Guoxiang Zou:** data curation (equal). **Xin Qi:** data curation (equal). **Jinxiu Meng:** formal analysis (equal), supervision (equal). **Xiaoran Huang:** methodology (equal), supervision (equal). **Juli Liu:** writing – original draft (equal), writing – review and editing (equal). **Haiwei He:** funding acquisition (equal), methodology (equal), writing – review and editing (equal). **Xin Li:** funding acquisition (equal), supervision (equal), writing – review and editing (equal).

## Ethics Statement

The study was conducted according to the guidelines of the Declaration of Helsinki and approved by the Ethics Committee of Guangdong Provincial People's Hospital (No. KY‐Z‐20210219‐02, date: 22 February 2021).

## Consent

Informed consent was obtained from all subjects involved in the study.

## Conflicts of Interest

The authors declare no conflicts of interest.

## Supporting information


Figures S1–S3.

## Data Availability

The data can be requested from the corresponding author.
