# Supplementary material for: Single‐Cell RNA‐Seq Reveals Injuries in Aortic Dissection and Identifies PDGF Signalling Pathway as a Potential Therapeutic Target
Source: J Cell Mol Med. 2024 Dec 25;28(24):e70293. doi: 10.1111/jcmm.70293 (PMC11669114; doi:10.1111/jcmm.70293)
Supplement: Supplementary file 1 — Figures S1–S3. [file JCMM-28-e70293-s001.pdf]

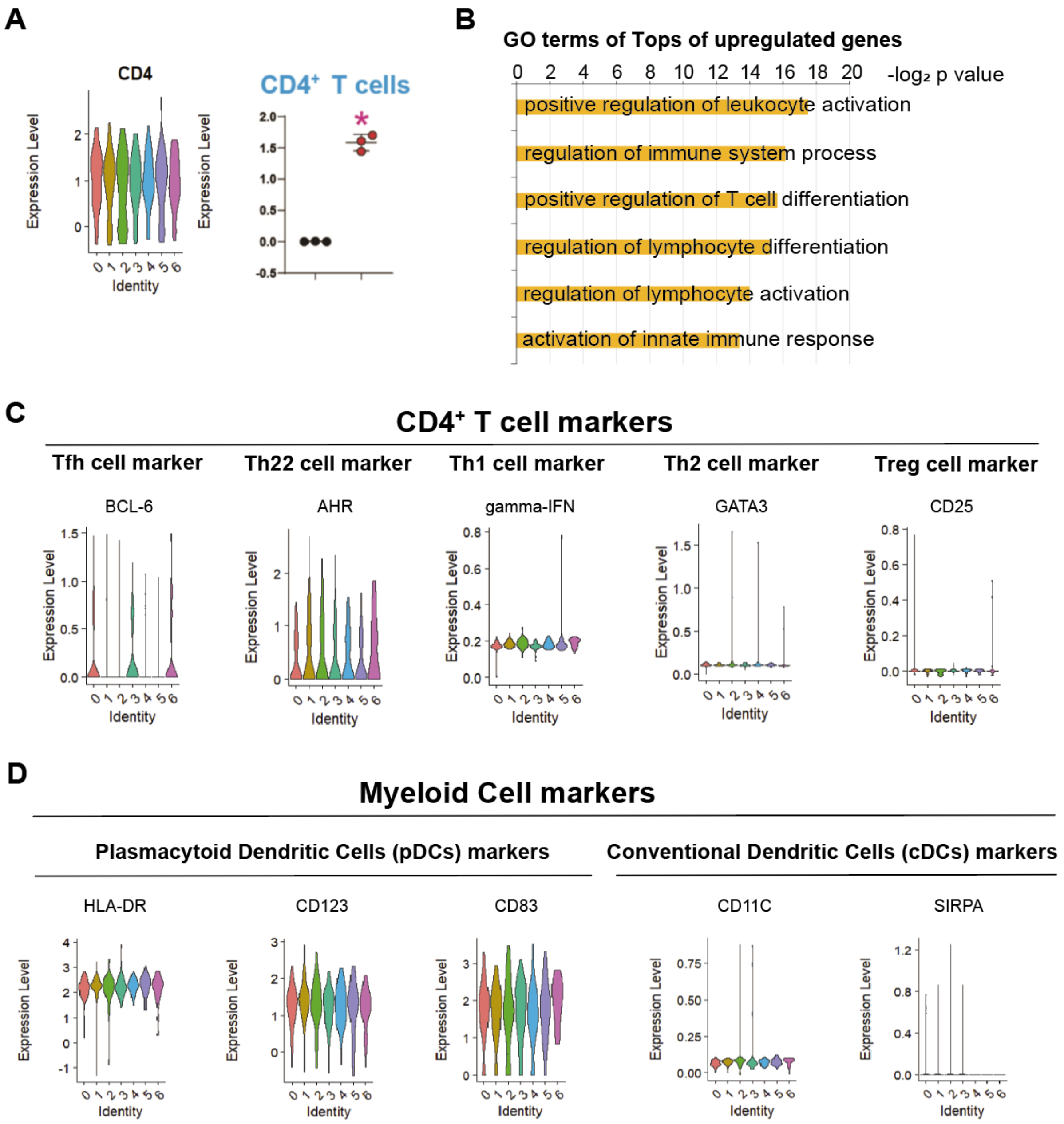

Supplemental Figure 1 Subcellular reclustering and signaling pathway enrichment analysis of CD4 immune T cells

(A) The expression level of CD4^{+} T cells.

(B) Gene ontology (GO) analysis indicated significant enrichment processes.

(C) The expression of typical CD4+ T cell markers.

(D) The expression of myeloid cell markers, such as those associated with Dendritic Cells (pDCs).

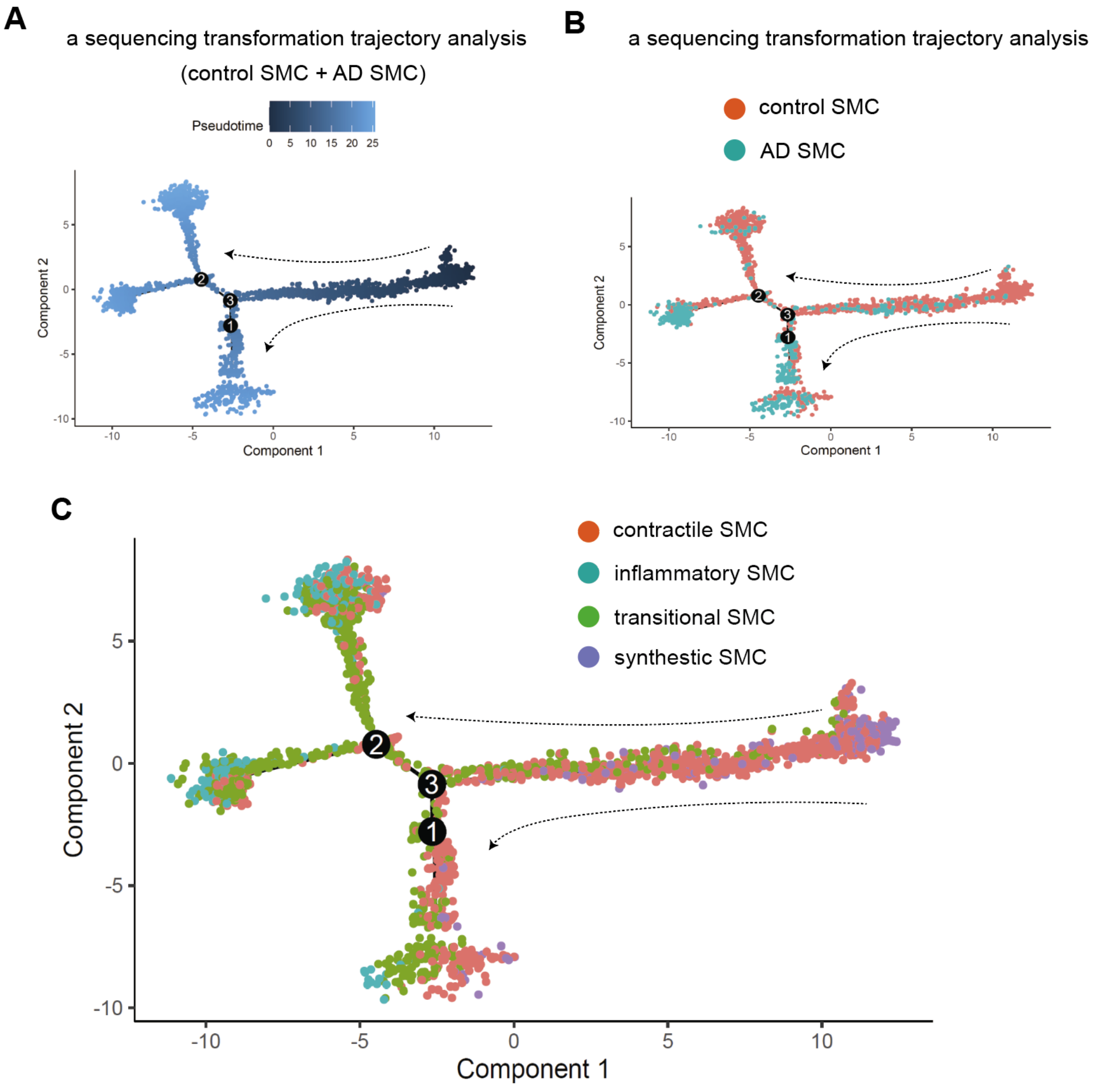

Supplemental Figure 2 Sequencing transformation trajectory analysis of SMC

(A) Sequencing transformation trajectory analysis of control-SMC and AD-SMC.

(B) The differences in cell transformation between AD-SMC and control-SMC.

(C) Detailed analysis based on gene expression profiles of different type of SMC.

GO biological process complete

events	P value	FDR
response to oxidative stress	2.20E-05	4.93E-04
detoxification of inorganic compound	3.34E-04	5.52E-03
detoxification	7.08E-04	1.05E-02
positive regulation of T cell mediated cytotoxicity	4.43E-03	4.68E-02

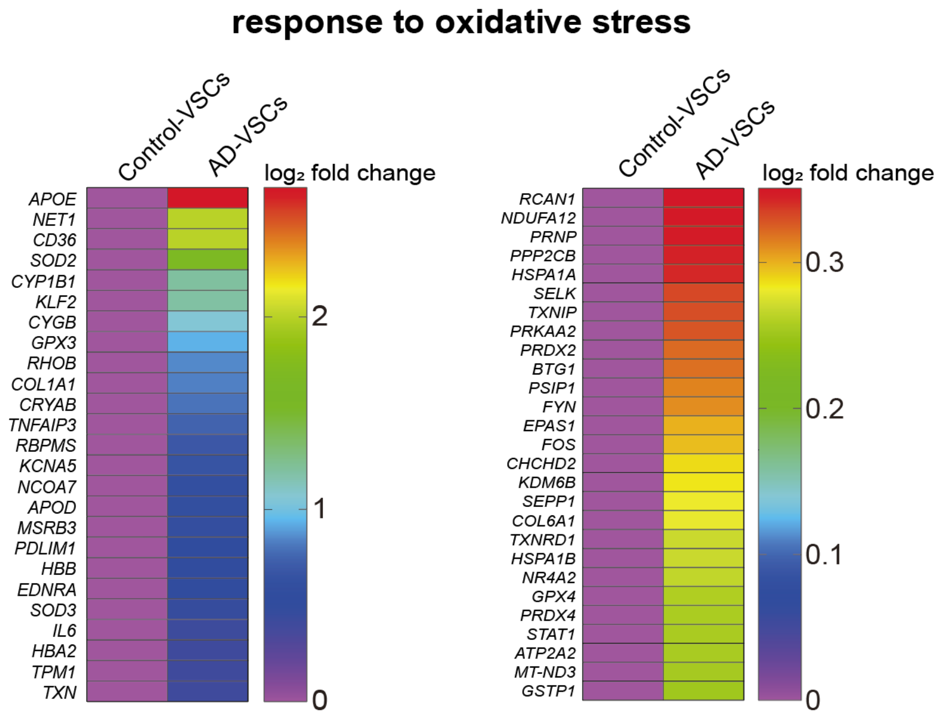

Supplemental Figure 3 The heat map of the expression levels of genes involved in oxidative stress events in AD
